# Structural equation modeling of university students’ academic resilience academic well-being, personality and educational attainment in online classes with Tencent Meeting application in China: investigating the role of student engagement

**DOI:** 10.1186/s40359-023-01366-1

**Published:** 2023-10-20

**Authors:** Yun Sun, Long Liu

**Affiliations:** School of Architecture & Artistic Design, Henan Polytechnic University, Henan, 454000 China

**Keywords:** Academic resilience, Academic well-being, Educational attainment, Personality, Student engagement, Undergraduate students

## Abstract

**Background:**

Online learning presents unique challenges for students, such as reduced social support and increased distractions. Understanding the psychological factors that contribute to educational attainment in online classes is therefore important.

**Purpose:**

This study aimed to investigate the structural relations among the psychological factors: academic resilience, personality, academic well-being, and educational attainment in online classes using the Tencent Meeting application in China. The study also explored the mediating role of student engagement in the relationship between the variables.

**Methodology:**

This study used structural equation modeling (SEM) to investigate the relationships among the variables of the study. The participants were 384 undergraduate, graduate, and postgraduate students from Henan Polytechnic University in China. The participants completed self-report surveys of academic resilience, academic well-being, educational attainment, student engagement and personality types.

**Data analysis:**

The data were analyzed using structural equation modeling (SEM) to examine the relationships among variables. The goodness of fit of the SEM was assessed using several fit indices, including the chi-square test, the comparative fit index (CFI), the Tucker-Lewis index (TLI), and the root mean square error of approximation (RMSEA). The study also conducted mediation analyses to explore the potential mediating roles of learner enjoyment in the relationships between psychological factors and educational attainment.

**Findings:**

The results of the study showed that all variables of the study were positively related to educational attainment. The findings suggest that promoting academic resilience, academic well-being, and student engagement may be effective strategies for enhancing educational attainment in online classes using the Tencent Meeting application in China.

**Conclusions:**

Using the Tencent Meeting application in China, this study provides insights into the complex interplay among several psychological factors and educational attainment in online classes. The findings highlight the importance of promoting academic resilience, personality, academic well-being, and student engagement to enhance educational attainment.

## Introduction

The rapid development of online learning platforms has become a significant trend in higher education worldwide. The COVID-19 pandemic has further accelerated the shift towards online learning, with many universities adopting online teaching and learning as the primary mode of instruction [1]. In response to this rapid change, it is crucial to examine the factors that contribute to students’ success in online classes. Academic resilience, student engagement, academic well-being, and personality type have been identified as important factors that contribute to students’ academic success in traditional classroom settings [2–5]. Student engagement reflects the degree of involvement, interest, and motivation students have in their learning [6]. Academic well-being encompasses students’ emotional and psychological well-being in relation to their academic experiences [7]. However, little research has been done on the role of these factors in online learning environments, particularly with respect to the Tencent Meeting application in China. Understanding the factors that contribute to academic success in online classes can inform the development of effective online learning strategies and support students’ academic achievement. Additionally, personality traits such as extraversion, openness, and conscientiousness have been found to be positively associated with academic achievement [8].

The rapid transition to online learning platforms in higher education has become a significant trend worldwide, and the COVID-19 pandemic has further accelerated this shift. Therefore, it is crucial to identify the factors that contribute to students’ academic success in online classes [1]. While academic resilience, learner enjoyment, and academic well-being have been identified as important factors in traditional classroom settings, their role in online learning environments is not yet fully understood [3–7]. Further research is needed to determine the extent to which these factors contribute to academic success in online classes, particularly in the context of the Tencent Meeting application in China. Additionally, the unique challenges posed by the pandemic, such as social isolation, financial difficulties, and health concerns, make it necessary to explore these factors to develop effective online learning strategies and support students’ academic achievement. Therefore, it is necessary to identify the factors that contribute to academic success in online classes and understand their role in the context of the pandemic and the Tencent Meeting application in China.

The rapid transition to online learning platforms in higher education has become a significant trend worldwide, and the COVID-19 pandemic has further accelerated this shift. Therefore, it is crucial to identify the factors that contribute to students’ academic success in online classes [1]. While academic resilience, learner enjoyment, and academic well-being have been identified as important factors in traditional classroom settings, their role in online learning environments is not yet fully understood [3–7]. Further research is needed to determine the extent to which these factors contribute to academic success in online classes, particularly in the context of the Tencent Meeting application in China. Additionally, the unique challenges posed by the pandemic, such as social isolation, financial difficulties, and health concerns, make it necessary to explore these factors to develop effective online learning strategies and support students’ academic achievement. Therefore, it is necessary to identify the factors that contribute to academic success in online classes and understand their role in the context of the pandemic and the Tencent Meeting application in China.

The present study aims to delve into the intricate interplay between various key factors affecting university students’ academic experience within the context of online education facilitated by the Tencent Meeting application in China. With the growing prevalence of online classes, understanding the dynamics that contribute to students’ academic resilience, academic well-being, and ultimately, their educational attainment has become crucial. By focusing on the mediating role of student engagement, this research seeks to bridge the gap in existing literature by shedding light on how individual traits interact with the virtual learning environment and impact students’ ability to adapt, persevere, and thrive academically. The findings hold the potential to provide educators, institutions, and policymakers with valuable insights into designing effective online learning strategies, support systems, and interventions tailored to the diverse personalities of students, ultimately enhancing the overall quality of online education in China and potentially beyond.

### Academic resilience, educational attainment

The concept of resilience has gained increasing attention with the emergence of positive psychology and positive organizational behavior [9, 10]. This trend is also observed in the university context, where research often focuses on disadvantaged groups and their connection to well-being [11]. Resilience is closely related to concepts like perseverance, grit, and coping, which have been extensively studied in educational settings. While both resilience and grit involve perseverance, resilience is characterized by a positive response and adaptation to challenges and adversity, whereas grit is defined as perseverance and passion for long-term goals [12].

Studies have shown that perseverance, coping, and grit are all linked to academic achievement [13, 14]. Resilience has been found to correlate with academic achievement in general student populations, including primary and secondary education [15]. Moreover, in a few studies investigating the relationship between resilience and academic achievement in tertiary education, resilience has been positively associated with factors such as grades and learning performance [16]. However, there is a lack of empirical research specifically exploring the link between resilience and study progress.

Personality traits, as represented by the Big Five model (neuroticism, extraversion, openness, agreeableness, and conscientiousness), have been extensively examined in relation to both resilience and academic achievement. Neuroticism tends to have a negative influence on study progress, while agreeableness, conscientiousness, and openness are generally associated with positive outcomes [14]. Openness, for instance, is related to cognitive intellect and the ability to analyze and understand experiences, which aids in positive adaptation and learning [17]. Conscientious students are known for their organizational skills and tendency to develop structured action plans when facing challenges [18]. Agreeableness and extraversion are associated with social skills and the establishment of social support networks, which contribute to resilience [19]. Emotional stability, characterized by being relaxed and self-confident, is positively related to resilience and the experience of positive emotions [20, 21]. Positive emotions have been found to broaden individuals’ mindsets and expand their repertoire of actions and resources, which aids in bouncing back from challenging conditions and maintaining focus on academic progress [4].

### Students’ personality and academic attainment

Students’ personality, defined as enduring patterns of thoughts, feelings, and behaviors, significantly influences their academic achievement [22]. The Big Five personality framework, encompassing Extraversion, Openness, Agreeableness, Conscientiousness, and Neuroticism, is commonly used to categorize personality traits [23]. Among these traits, Conscientiousness and Openness have demonstrated particular relevance to academic achievement [24]. Conscientiousness involves self-regulation, achievement-striving, and organization. High levels of Conscientiousness are associated with greater effort investment in homework and fewer counterproductive academic behaviors [25]. Consistently, Conscientiousness exhibits a correlation of approximately 0.25 with academic achievement across diverse studies and educational contexts [26].

Openness is linked to adaptive learning approaches and motivation. Individuals high in Openness actively seek out learning opportunities and prefer intellectually stimulating environments, contributing to their intellectual development [27]. Meta-analytic findings indicate a modest correlation of around 0.12 between Openness and academic achievement at the secondary school level. The impact of the remaining Big Five traits on academic achievement is less consistent. Higher levels of Neuroticism can lead to anxiety and worry, potentially diverting attention from academic tasks [28]. Extraversion, although it may provide energy for learning, can also result in distractions and a preference for socializing over studying [29]. Agreeableness may benefit academic performance due to cooperative behavior and compliance with instructions [29]. However, the associations between these traits and academic achievement tend to be nonsignificant or close to zero. Considering the bidirectional relationship between academic achievement, intelligence, and personality is crucial. Academic success or failure can influence the development of identity and personality maturity in adolescents, potentially reinforcing achievement-related behaviors and personality traits such as Conscientiousness and Openness [30].

### Students’ engagement and personality types

Research has predominantly focused on engagement as a predictor of objective outcomes such as achievement and grades rather than on engagement itself as the outcome. Given the negative relations found between engagement and outcomes such as burnout [31], there is a pressing need for research that directly examines the individual differences correlates of engagement. Hence, the present study explores the role of students’ “Big-5” personality traits [32] on their engagement. Big-5 or “the Five Factor Model” of personality [FFM] has been accepted as the dominant model for categorizing individual differences in personality [33]. The FFM suggests that individual differences in behavior should be classified in terms of five independent traits, namely extraversion, agreeableness, conscientiousness, emotional stability, and imagination [34].

The role of personality in academic achievement is well-documented [35]. In particular, conscientiousness has consistently and positively been correlated with exam and essay performance [36], whereas neuroticism has been found to be a negative predictor of academic performance [37] and examination performance [38]. Academic performance more generally has been associated with agreeableness, conscientiousness, and openness to experience [25]. Although the literature has yielded ambiguous results in regard to extraversion [39], the relationship between FFM and achievement is relatively well-documented [25]. Nevertheless, the role of the FFM in student engagement has yet to be studied in depth. Additionally, previous research has typically examined the role of personality on proxies of engagement such as retention [40], academic performance [41], and learning approaches [42]. Support comes from a study by Komarraju and Karau [43] who reported that the traits of extraversion and openness to experience were related to engagement. Limited research has found a link between agreeableness and engagement per se [44], but this does not appear to have been replicated, possibly due to differences in the operationalization of engagement [45]. Interestingly, research suggests that agreeableness may be related to the emotional regard a student has toward studying. Critically, these findings indicate the importance of examining a multidimensional model of engagement. In an effort to enhance our theoretical understanding of the nature of engagement, the present study examines the relationship between personality and a multidimensional model of engagement.

### Students’ engagement and educational attainment

Students’ engagement is a crucial aspect to consider when assessing teaching in a digital environment [46]. While some researchers define engagement based on perspectives and principles regarding the importance of learning, others view it as going beyond the minimum effort required [46]. An analysis of digital learning publications revealed that approximately half of the studies mentioned the term “engagement” [47]. However, the research directly linking engagement to digital learning is limited and exceptional in some cases.

Several scientists have proposed a multidimensional model of engagement [48], which encompasses behavioral, emotional, and cognitive aspects [49]. Behavioral engagement relates to involvement, persistence, and participation in academic activities [46]. Emotional engagement focuses on positive and negative reactions to peers, professors, institutions, and the evaluation of learning outcomes. Cognitive engagement involves students’ investment and understanding of the topic, encompassing thoughtfulness and willingness to exert effort to comprehend complex ideas and master difficult skills [46].

The academic engagement has numerous long-term positive effects, including pursuing higher education, maintaining consistent learning patterns, improving job prospects, fostering positive self-perception and well-being, and reducing depressive symptoms [50, 51]. Thus, participation in academic activities can yield positive and wide-ranging outcomes beyond the educational setting. The academic engagement has also been found to be strongly associated with academic motivation and performance, as students who engage in academic activities tend to rate their studies higher, achieve higher scores, and report lower levels of academic disengagement and avoidance [51].

Log records from electronic learning management systems can provide independent and related data, such as the frequency of student logins. While log files have been studied for various purposes, their use in analyzing interactions and engagement has been relatively scarce. Recently, researchers such as Gobert, Baker, and Wixon [52] have developed procedures to identify engagement in online learning environments for investigating scientific questions. However, alternative methods like interviews have their advantages. Interviews can provide comprehensive information about why students choose to participate or not in specific events, why there are variations in communication performance among students, and the contextual factors that may influence student engagement or disengagement [53]. Considering that each method of measurement has its strengths and weaknesses, it is advisable to employ multiple techniques to assess students’ engagement, as recommended by several academics [54]. It is worth noting that the availability of an electronic educational platform, including necessary resources and a user-friendly interface, plays a significant role in motivating students and enhancing their engagement. An electronic educational platform that offers convenience and engaging content can effectively promote student engagement. Engagement has garnered attention in recent years as a key factor in academic success. Positive emotions are believed to indirectly influence academic outcomes through motivational processes like engagement [55]. Engagement is closely associated with motivational processes and is considered a crucial factor in achieving academic goals. Interested students tend to invest more effort in academic tasks, leading to successful task completion and improved academic performance. In work environments, engagement is described as a state of mind characterized by energy, dedication, and absorption [56]. Energy reflects high levels of mental activity during work, dedication refers to a sense of worth, enthusiasm, inspiration, pride, and challenge, while absorption entails complete concentration and satisfaction with one’s work, causing time to pass quickly. This concept has also been applied in a scholarly context, focusing on students’ tasks and activities [56]. Engaged students feel energized, passionate about their studies, and actively involved in their academic life [56]. Empirical evidence supports the notion that engaged university students perform better academically [57]. Experimental designs have also demonstrated a positive relationship between engagement and academic performance [57].

### Students’ academic resilience and engagement

Nurmala et al. [58] emphasized that the uncertainty associated with changes in learning methods can act as a stressor for students [58]. This has been evident during the Covid-19 pandemic, where students have experienced academic stressors due to shifts in learning methods, leading to decreased learning motivation and reduced student engagement [59]. Continuous transitions and uncertainties pose challenges for students and can negatively impact their engagement in the learning process. To navigate these changes and challenges, students require resilience, as it enables them to skillfully adapt and cope with academic stress [60, 61].

Resilience plays a significant role in enhancing the quality of education and overall personal growth across various disciplines [62]. It is a dynamic characteristic that can change over time as individuals interact with their environment and undergo personal development [63]. Resilience encompasses three fundamental aspects: the ability to adapt and change as needed, the capacity to bounce back quickly from challenges or setbacks, and the capability to maintain confidence and strength in the face of change [64].

In higher education, students are expected to take responsibility for building their academic skills independently. However, many students still rely on others in their educational journey. Students encounter various problems and difficulties related to their academics, and their ability to effectively handle these challenges varies. Students who struggle to solve problems may experience negative emotions and be more susceptible to stress. It is crucial for students to recognize their inner capabilities, take ownership of their academic responsibilities, and develop problem-solving skills. Academic resilience serves as a protective factor that equips students to survive in difficult conditions, overcome adversity, and adapt positively to academic pressures and demands [63].

Sari et al. [65] explain that academically resilient students effectively navigate setbacks, challenges, adversity, and pressure within an academic context. Resilient students demonstrate a positive attitude when faced with obstacles [65]. Research supports the notion that individuals with resilience exhibit positive emotions when confronting various situations. In the post-pandemic era, it is essential for students to adapt to new conditions, overcome unwanted situations, and effectively address learning challenges, ultimately becoming problem solvers in their educational journey [66].

Academic resilience is characterized by a student’s ability to withstand academic difficulties and maintain optimism, positive thinking, and problem-solving skills. It reflects an individual’s strength and tenacity to overcome negative emotional experiences in the face of significant obstacles encountered during the learning process [67, 68]. Students with high academic resilience embrace challenges as opportunities to prove themselves as active learners in the college environment [69].

### Academic Well-being, and educational attainment

Academic well-being is a holistic measure of students’ academic experiences, including their sense of belonging, satisfaction with school, and engagement in learning. It has been identified as an important factor that contributes to students’ academic success [70]. Educational attainment refers to the level of education that an individual has achieved and is often used as a measure of academic success. This review of literature aims to explore the relationship between academic well-being and educational attainment and to identify the factors that contribute to this relationship. Academic well-being seems to be positively associated with educational attainment. A study by Suldo, Shaunessy, and Hardesty [71] found that students who reported higher levels of academic well-being, including positive emotions, high academic self-concept, and supportive relationships, also had higher grades and test scores. Similarly, a study by Salmela-Aro and Upadyaya [72] found that students who reported higher levels of academic well-being were more likely to complete secondary education and pursue higher education. Several factors have been identified as contributing to the relationship between academic well-being and educational attainment. One of the primary factors is social support, or the extent to which students feel supported by parents, teachers, and peers in their academic pursuits. Students who have high levels of social support are more likely to develop academic well-being and achieve academic success [70]. Additionally, a positive school climate, characterized by high expectations, supportive relationships, and opportunities for engagement, has been found to promote academic well-being and educational attainment [70]. Furthermore, student motivation, particularly intrinsic motivation, has been found to be positively associated with academic well-being and educational attainment [71]. Finally, academic self-concept, or the belief in one’s academic abilities, has been found to be a critical factor that contributes to academic well-being and educational attainment [73, 74].

### Well-being and engagement

The transition to online learning presents challenges for students’ well-being and engagement, leading to a decline in their academic performance [75]. Student engagement is influenced by social interactions in various learning spaces, including interactions with peers and academic staff. However, the shift to online learning during the pandemic posed difficulties for both students and academic staff in adapting to this new context [75]. Research has extensively examined the impact of the pandemic on students’ well-being and engagement, highlighting the alarming decrease in student engagement across academic contexts [76, 77]. In fact, a significant number of students have reported that low engagement during online lectures negatively affected their learning experience [75]. It is crucial to recognize the importance of student engagement in online learning, as emphasized by various studies [78–80]. As universities increasingly offer online and hybrid courses, there is a growing need for research that investigates student engagement in the online learning environment.

**Fig. 1:**
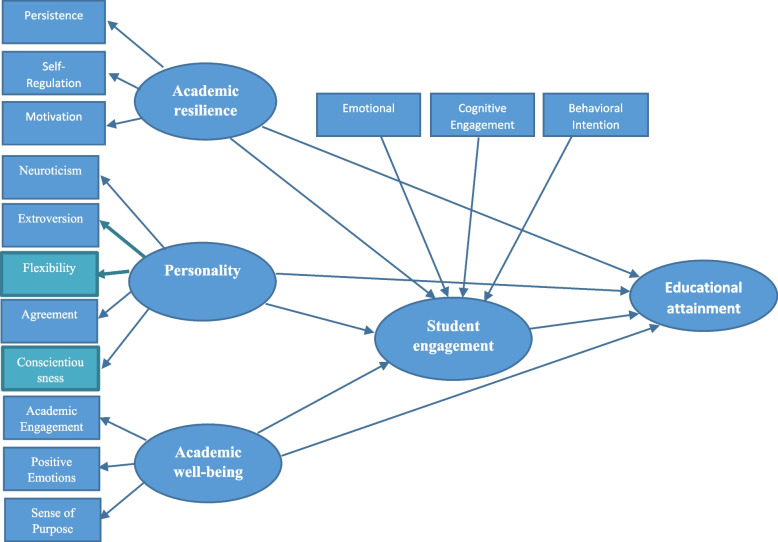
Hypothetical structural modeling of the dependent, independent, and moderating variables

Academic engagement is a complex and multidimensional concept that defies a single definition [81]. It has been examined using various international scales and from different perspectives [80]. Academic engagement is considered a key factor influencing learning and academic success in the university context. According to Marôco et al. [82], student engagement is associated with physical and psychological energy during educational experiences. High levels of engagement contribute to positive well-being, reduced dropout rates, and lower risk of burnout [82]. It is also linked to student self-efficacy [83]. Engaged students demonstrate a sense of purpose, persistence, resilience, and emotional connection to others in learning environments, experiencing a sense of belonging and high self-efficacy [84]. Therefore, student engagement is a dynamic and multidimensional construct that evolves over time within an individual’s self-determination [85]. Importantly, interventions can positively influence student engagement, leading to improved performance and reduced dropout rates [85]. Based on the above-mentioned related studies, the conceptual model is given in Fig. 1.

In line with the above hypothetical framework, the following hypotheses were stated:

- H1 = The structural model of academic resilience, personality, and academic well-being on educational attainment with the mediating variable of learner has goodness of fit.
- H2 = Academic resilience has a significant effect on educational attainment.
- H3 = Academic resilience has a significant effect on student engagement.
- H3 = Academic resilience has a significant indirect effect on educational attainment through the mediating variable of engagement.
- H4 = Students’ personality has a significant effect on educational attainment.
- H5 = Students’ personality has a significant effect on student engagement.
- H6 = Students’ personality has a significant indirect effect on educational attainment through the mediating variable of engagement.
- H7 = Students’ well-being has a significant effect on educational attainment.
- H8 = Students’ well-being has a significant effect on student engagement.
- H9 = Students’ well-being has a significant indirect effect on educational attainment through the mediating variable of engagement.
- H10 = Student engagement has a significant effect on the student’s educational attainment.

## Methodology

The research method chosen for this study was a cross-sectional survey design with structural equation modeling (SEM) analysis. A sample of students from a selected educational institution participated in the study. The survey was administered to the students, who were asked to complete a set of self-report measures that assessed academic resilience, learner enjoyment, academic well-being, and educational attainment. The cross-sectional survey design allowed the researchers to gather data from the student population at a specific point in time, providing insights into the relationships between the variables under investigation. The study aimed to explore the factors that contributed to academic resilience, learner enjoyment, academic well-being, and educational attainment among the students. To collect the necessary data, the researchers administered a survey to the selected students. The survey comprised a series of self-report measures carefully selected based on established scales and existing literature to ensure reliability and validity. After completing the data collection, the researchers utilized structural equation modeling (SEM) analysis to examine the relationships between the variables of interest. SEM is a statistical technique that allows for the assessment of complex relationships among variables, considering both direct and indirect effects. By employing SEM, the researchers tested the hypothesized relationships and assessed the overall fit of the proposed model.

### Participants

The sample for this study was drawn from Henan Polytechnic University, which is a comprehensive university in China. The sample included participants from different levels of education, such as undergraduate, graduate, and postgraduate programs. The selection of participants was based on a random sampling technique that ensured that the sample was representative of the overall student population at the university. The sample size for this study was determined based on the power analysis and sample size calculation. The power analysis was conducted to estimate the minimum sample size required to detect statistically significant differences in the study’s outcomes, with a specified level of significance and power. The sample size calculation took into account the effect size, which was based on previous research on similar topics, and the level of significance, which was set at 0.05. The power of the study was set at 0.80, which is a commonly used level of power in social science research. In total, 384 participants were recruited for the study. Of these, 195 were male (50.8%) and 189 were female (49.2%). The age of participants ranged from 18 to 55 years, with a mean age of 22.8 years (SD = 3.5). The majority of participants were in the age range of 18–20 years (33.3%), followed by 21–23 years (29.9%), and 24–26 years (21.9%). A smaller proportion of participants were in the age ranges of 27–29 years (9.1%) and 30 years and above (5.7%).

In terms of education level, the majority of participants were enrolled in undergraduate programs (58.9%), followed by graduate programs (29.9%), and postgraduate programs (11.2%). The sample included participants from a variety of academic disciplines, including engineering, business, humanities, social sciences, and natural sciences. The diversity of the sample in terms of gender, age, and education level ensures that the study’s findings are generalizable to a broader population of young adults in China. The demographic profile of the participants is presented in Table 1.

**Table 1:** Demographic profile of the participants

	Number	Percent
Gender		
Male	195	50.8%
Female	189	49.2%
Age		
18–20	128	33.3%
21–23	115	29.9%
24–26	84	21.9%
27–29	35	9.1%
30 and above	22	5.7%
Level		
Undergraduate	226	58.9%
Graduate	115	29.9%
Postgraduate	43	11.2%

### Measures

Different instruments were used in this study, each is explained as follows :

- ***Academic Resilience Scale (ARS)***
  The first instrument was the ARS which is a self-report measure that assesses students’ ability to overcome academic adversity, including challenges such as low grades and academic setbacks. The ARS includes items that assess students’ persistence, self-regulation, and motivation to succeed academically. The scale measures three main dimensions: Persistence, Self-regulation, and Motivation. The total score ranges from 20 to 100, with higher scores indicating higher levels of academic resilience [86].
- ***Academic Well-being Scale***
  The second instrument is the AWS which is a self-report measure that assesses students’ overall academic well-being. The AWS measures four main dimensions: Academic Engagement, Positive Emotions, Sense of Purpose, and Life Satisfaction. The total score ranges from 20 to 100, with higher scores indicating higher levels of academic well-being [87].
- ***Student engagement***
  The third instrument was student engagement which is a self-report measure that assesses the extent to which students are engaged in classroom activities. The scale measures three dimensions of engagement: Affective Enjoyment, Cognitive Engagement, and Behavioral engagement. The total score ranges from 12 to 60, with higher scores indicating higher levels of engagement [88].
- ***Personality indicator***
  The fourth instrument was the 5-factor personality indicator [89]. It measures an individual’s personality traits across five broad domains: Openness, Conscientiousness, Extraversion, Agreeableness, and Neuroticism. In this study, educational attainment was limited Grade Point Average (GPA). In Chinese higher education Grade Point Average (GPA) is calculated based on the grades received in courses. The GPA scale typically ranges from 0 to 4.0, with 4.0 being the highest possible GPA.

### Data collection

Data were collected through a survey questionnaire that included the selected self-report measures for academic resilience, academic well-being, student engagement, educational attainment, and personality type indicator scale. The survey questionnaire was administered electronically, and the students were invited to complete the survey during a designated time period. The data were collected in January, 2023. To ensure the privacy and anonymity of the participants, the survey was designed to be anonymous, and the students were assured of the confidentiality of their responses. This approach aimed to encourage honest and candid responses from the students, as they could provide their feedback without concerns about potential identification or repercussions. The survey administration process involved sending electronic invitations to the selected students and providing them with instructions and a link to access the survey. The students were informed about the purpose of the study and the importance of their participation in contributing to the research. They were also given details about the estimated time required to complete the survey and any potential incentives, such as gift cards or participation certificates, for their involvement.

During the designated time period, the students had the opportunity to complete the survey at their convenience, using their own electronic devices with internet access. They were encouraged to respond to the survey questions honestly and thoughtfully, providing their perspectives and experiences related to academic resilience, learner enjoyment, academic well-being, and educational attainment. By utilizing an electronic survey administration method, the researchers aimed to streamline the data collection process and facilitate efficient data entry and analysis. Electronic surveys also reduce the potential for errors in data transcription and ensure that the responses are securely stored for analysis. The returned questionnaires with missing data were excluded from final analysis.

### Data analysis

The collected data were subjected to rigorous statistical analysis to examine the relationships between the variables of interest, namely academic resilience, student engagement, academic well-being, educational attainment, and personality type. The following steps were undertaken to analyze the data. First, data cleaning was performed to ensure the completeness and accuracy of the collected survey responses. Missing or erroneous data points were addressed through appropriate techniques such as imputation or removal, maintaining the integrity of the dataset. Descriptive statistics were then calculated to provide a summary of the key variables. Measures such as means and standard deviations were computed to describe the central tendency, variability, and distribution of the data. Reliability analysis was conducted to assess the internal consistency of the self-report measures. Measures such as Cronbach’s alpha coefficient were computed for each scale used in the survey to evaluate their reliability and consistency. Bivariate correlation analysis was performed to examine the relationships between the variables of interest. Pearson’s correlation coefficient or other appropriate correlation measures were computed to determine the strength and direction of the associations among the variables. To test the hypothesized relationships among the variables, structural equation modeling (SEM) was employed. The SEM analysis allowed for the assessment of both direct and indirect effects, providing a comprehensive understanding of the complex relationships. Model fit indices such as chi-square, comparative fit index (CFI), root mean square error of approximation (RMSEA), and standardized root mean square residual (SRMR) were evaluated to assess the adequacy of the proposed model. Subgroup analyses were conducted to explore potential variations in the relationships among different demographic groups.

### Ethical considerations

The studies involving human participants were reviewed and approved by Institutional Review Board of School of Architecture and Artistic Design, Henan Polytechnic University. The participants provided their written informed consent to participate in this study. The study was conducted in accordance with the Declaration of Helsinki. Anonymity and confidentiality were prioritized, and data were securely stored. The research was conducted in accordance with the ethical guidelines of the selected educational institution and relevant research ethics committee.

## Results

The results of the study are presented in two sections: evaluating the model and results for investigating the role of the student’s personality types.

### Evaluation of the hypothetical model

**Fig. 2:**
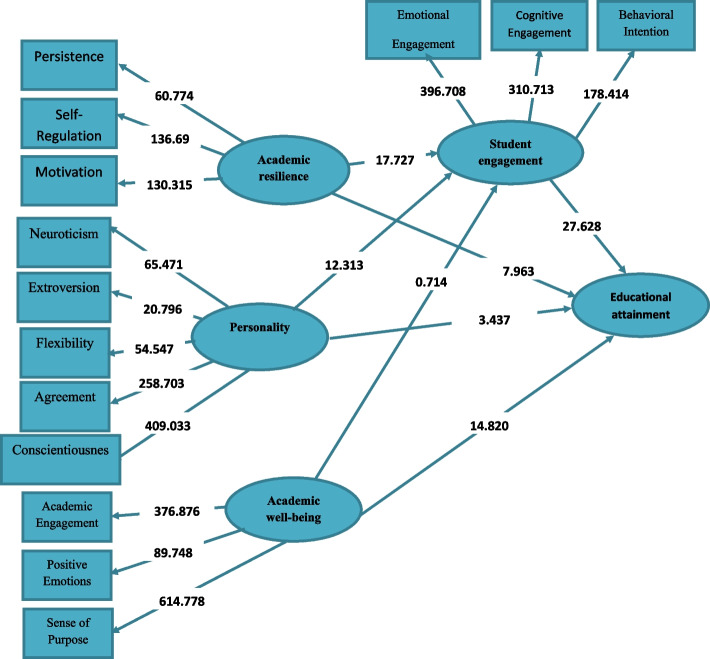
Empirical structural model (path) and measurement of educational attainment for students based on non-standard coefficients (significant coefficients)

**Fig. 3:**
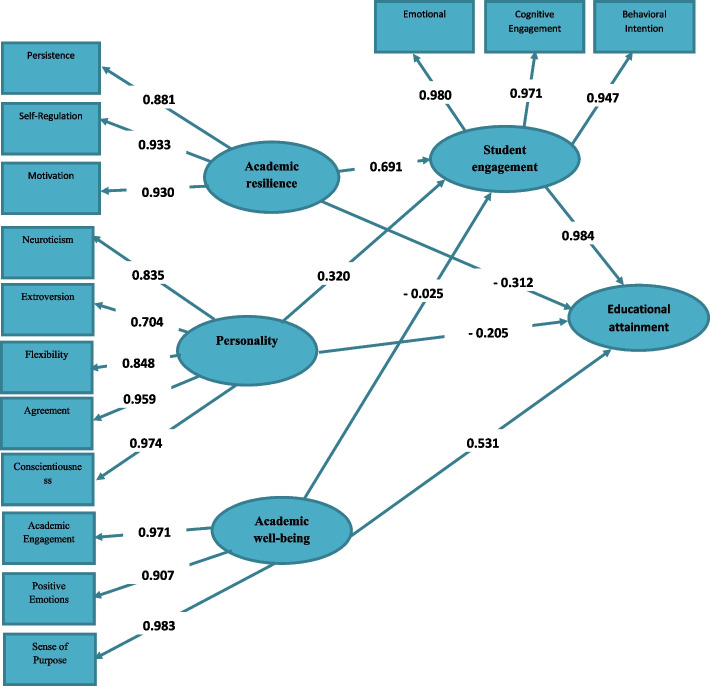
Empirical structural model (path) and measurement of educational attainment for students based on standard coefficients (effect coefficients)

To test the general hypothesis of the research, a multivariate analysis and structural equation modeling using Smart-PLS3 software was used due to the non-normal distribution of data and the low sample size. The results of the model in displaying non-standard coefficients (significant coefficients) and standard coefficients (effect coefficients) are shown in Figs. 2 and 3, respectively.

In general, the assessment of structural equation models using Smart-PLS3 software involves the examination of three models: the outer model, the inner model, and the overall empirical model. The outer model, which corresponds to the measurement model in structural equation modeling, depicts the relationships between latent or unobserved variables (independent and dependent) and observed or manifest variables (in this case, indicators). The inner model corresponds to the structural model or path analysis in structural equation modeling and explores the relationships between latent or unobserved variables. Additionally, the overall empirical model evaluates the overall fit of the model. Subsequently, the evaluation and testing of the empirical structural model (path) and measurement, as well as the overall empirical model of educational attainment for students, were conducted. The results for the measurement model (outer) are presented in Table 2.

**Table 2:** Validity and reliability of the student educational attainment model

**Construct**	**Indicator**	**Validity assessment**			**Reliability assessment**		
		**convergent validity**		**divergent validity**			
		FL	AVE	Fornell & Larker Index	Cronbach’s alpha	Rho-A	Composite Reliability
Academic resilience	Persistence	0.881	0.838	0.915	0.903	0.911	0.939
	Self-Regulation	0.933					
	Motivation	0.930					
Personality	Neuroticism	0.835	0.756	0.869	0.915	0.939	0.939
	Extroversion	0.704					
	Flexibility	0.848					
	Agreement	0.959					
	Conscientiousness	0.974					
Academic well-being	Academic Engagement	0.971	0.911	0.954	0.950	0.956	0.968
	Positive Emotions	0.907					
	Sense of Purpose	0.983					
Student engagement	Emotional	0.980	0.933	0.966	0.964	0.964	0.977
	Cognitive Engagement	0.971					
	Behavioral Intention	0.947					

As shown in Table 1 and Fig. 3, the cross-loadings of the independent and mediating variables, namely Academic Resilience, personality, and Academic Well-being, on educational attainment, with the mediation of The Learner Enjoyment, were all above the acceptable threshold of 0.7 and statistically significant at the 0.01 level (*p* < 0.01), indicating acceptable relationships between the observed variables (components) and the latent variables (independent and mediating variables). Additionally, the extracted Average Variance Extracted (AVE) values for all research constructs were above the acceptable threshold of 0.5, indicating convergent validity for the constructs. Furthermore, the discriminant validity (distinctiveness) assessment, as demonstrated in Table 1, indicated that the square root of the AVE of each construct was greater than its correlation with other constructs, signifying that the latent variables (independent and mediating variables) had a stronger association with their own components than with other constructs, which confirms the acceptable discriminant validity of the model. Finally, the internal consistency (reliability) assessment, as evidenced by the results in Table 1, revealed that Cronbach’s alpha coefficient for the research constructs (independent and mediating variables) was above the acceptable threshold of 0.7. Moreover, the Composite Reliability (CR) and the homogenous Rho-A coefficient were also above the acceptable threshold of 0.7 for all constructs, indicating acceptable composite reliability for the constructs.

### Internal model evaluation (structural or path model)

#### Path coefficients of the structural model (significance coefficients)

In this section, all the paths shown in the internal model (relationships between constructs based on research hypotheses) are evaluated based on the T-test for significance. Therefore, if the test confidence level is above 96.1 and 58.2, they will be confirmed statistically at the 95% and 99% confidence levels, respectively. As shown in Fig. 2, all hypotheses have been accepted, and their T-values are statistically significant at the 99% and 95% confidence levels (0.01*P* <) and (0.05*P* <), respectively.

#### Examination of the coefficient of determination

The coefficient of determination, which indicates the total explained variance of the dependent variable (Educational attainment) based on independent and mediating variables in the structural model, is equal to 0.983, which is at a very good level. In addition, the independent variables of the study have been able to explain and predict 96.1% of the variance of the mediating variable.

#### Examination of research hypotheses

The stated hypotheses were tested results are presented in Table 3.

**Table 3:** Evaluation of the stated hypotheses

Research hypotheses	Effect sizes			Results
	Non-standard coefficients		Standard Coefficients	
	**T**	***p***		
The structural model of academic resilience, personality, and academic well-being on educational attainment with the mediating variable of learner has goodness of fit.	21.070	*P* < 0/01	0.778	accepted
H2 = Academic resilience has a significant effect on educational attainment.	9.823	*P* < 0/01	0.145	accepted
H3 = Academic resilience has a significant effect on student engagement.	1.941	*P* < 0/05	0.067	accepted
H3 = Academic resilience has a significant indirect effect on educational attainment through the mediating variable of engagement.	3.711	*P* < 0.01	0.677	Accepted
H4 = Students’ personality has a significant effect on educational attainment.	21.070	*P* < 0/01	0.778	accepted
H5 = Students’ personality has a significant effect on student engagement.	9.823	*P* < 0/01	0.145	Accepted
H6 = Students’ personality has a significant indirect effect on educational attainment through the mediating variable of engagement.	9.104	*P* < 0/01	0.413	Accepted
H7 = Students’ well-being has a significant effect on educational attainment.	1.021	*P* > 0/05	0.028	rejected
H8 = Students’ well-being has a significant effect on student engagement.	14.040	*P* < 0/01	0.544	Accepted
H9 = Students’ well-being has a significant indirect effect on educational attainment through the mediating variable of engagement.	28.535	*P* < 0/01	0.939	Accepted

Finally, based on the overall evaluation of the model according to the RNS Theta index, which has a value of 0.623 out of 100, it can be concluded that the generated model is relatively similar to the theoretical model. Therefore, the general hypothesis of the research, which is based on the structural and measurement model of the effects of academic resilience, and academic well-being on the educational attainment of students with the mediating variable of Learner Enjoyment, is confirmed.

## Discussion

The first finding of the study is that AR has a significant on the student’s educational attainment. It was also found that personality and academic resilience are significantly correlated. Therefore, it can be strongly argued that resilience, perseverance, grit, and coping are all linked to academic achievement [12–14]. Resilience, in particular, has been found to correlate positively with academic achievement in primary, secondary, and tertiary education [15, 16]. It can also be argued that neuroticism tends to have a negative influence on study progress, while agreeableness, conscientiousness, and openness are generally associated with positive outcomes [14, 17–19]. Emotional stability is positively related to resilience and the experience of positive emotions, which have been found to broaden individuals’ mindsets and expand their repertoire of actions and resources, aiding in bouncing back from challenging conditions and maintaining focus on academic progress [4, 20, 21].

The next finding was that the students’ personality has a significant impact on academic achievement and this finding highlights the importance of considering the Big Five personality traits, particularly Conscientiousness and Openness. The findings suggest that Conscientiousness is associated with greater effort investment in homework and fewer counterproductive academic behaviors, which leads to higher academic achievement [25]. Openness is linked to adaptive learning approaches and motivation, which contribute to intellectual development and academic success [27]. However, the impact of other personality traits, such as Neuroticism, Extraversion, and Agreeableness, on academic achievement is less consistent [28, 29]. It is important to note that personality traits and academic achievement have a bidirectional relationship. Academic success or failure can influence the development of identity and personality maturity in adolescents, potentially reinforcing achievement-related behaviors and personality traits such as Conscientiousness and Openness [30]. This highlights the importance of creating a supportive environment that promotes academic success and the development of positive personality traits.

The findings also suggest that students’ engagement is a crucial factor in their educational attainment [46–57]. Engagement has been defined in various ways, including as involvement beyond the minimum effort required and as a multidimensional construct encompassing behavioral, emotional, and cognitive aspects [46–49]. Academic engagement has numerous long-term positive effects beyond the educational setting, including fostering positive self-perception and well-being, reducing depressive symptoms, and improving job prospects [50]. Additionally, academic engagement has been found to be strongly associated with academic motivation and performance, as students who engage in academic activities tend to rate their studies higher, achieve higher scores, and report lower levels of academic disengagement and avoidance [51].

Various methods have been used to measure engagement, including electronic learning management systems, log files, and interviews [52–54]. While each method has its strengths and weaknesses, it is advisable to employ multiple techniques to assess students’ engagement. The availability of an electronic educational platform with necessary resources and a user-friendly interface plays a significant role in motivating students and enhancing their engagement [47]. Engagement is closely associated with motivational processes and is considered a crucial factor in achieving academic goals [55]. Engaged students feel energized, passionate about their studies, and actively involved in their academic life [56]. Empirical evidence supports the notion that engaged university students perform better academically [57]. Experimental designs have also demonstrated a positive relationship between engagement and academic performance [57]. Positive emotions are believed to indirectly influence academic outcomes through motivational processes like engagement [55]. Therefore, promoting engagement in academic activities is essential for achieving academic success.

The findings suggest that students’ academic resilience has a significant effect on their engagement in the learning process [58–69]. The uncertainty associated with changes in learning methods can act as a stressor for students, leading to decreased learning motivation and reduced student engagement [58, 59]. Continuous transitions and uncertainties pose challenges for students and can negatively impact their engagement in the learning process. To navigate these changes and challenges, students require resilience, as it enables them to skillfully adapt and cope with academic stress [60, 61]. Resilience plays a significant role in enhancing the quality of education and overall personal growth across various disciplines [62]. It is a dynamic characteristic that can change over time as individuals interact with their environment and undergo personal development [63]. Resilience encompasses the ability to adapt and change as needed, the capacity to bounce back quickly from challenges or setbacks, and the capability to maintain confidence and strength in the face of change [64].

In higher education, students are expected to take responsibility for building their academic skills independently. However, many students still rely on others in their educational journey. Academic resilience serves as a protective factor that equips students to survive in difficult conditions, overcome adversity, and adapt positively to academic pressures and demands [63]. Resilient students demonstrate a positive attitude when faced with obstacles and exhibit positive emotions when confronting various situations [65]. Academic resilience is characterized by a student’s ability to withstand academic difficulties and maintain optimism, positive thinking, and problem-solving skills. It reflects an individual’s strength and tenacity to overcome negative emotional experiences in the face of significant obstacles encountered during the learning process [67, 68]. Students with high academic resilience embrace challenges as opportunities to prove themselves as active learners in the college environment [69].

The research findings draw attention to the profound impact of students’ well-being on both their level of engagement and overall educational attainment [75–85, 90]. This relationship becomes particularly salient in the context of the unprecedented shift to online learning that was prompted by the pandemic. This transition posed significant challenges for students and educators alike, as they grappled with adapting to this novel educational landscape, ultimately leading to a notable decline in academic performance [75].

Central to this dynamic is the pivotal role of student engagement, which is widely recognized as a cornerstone of learning and academic success within the university setting [82]. Robust engagement has far-reaching implications, contributing not only to improved academic outcomes but also to positive well-being, reduced dropout rates, and a decreased risk of burnout [82]. Extensive research has delved into the repercussions of the pandemic on students’ well-being and their level of engagement, revealing an alarming decline in student engagement across various academic contexts [76, 77]. In this rapidly evolving educational environment, it has become imperative to acknowledge the significance of nurturing student engagement, especially in the realm of online and hybrid courses that universities are increasingly offering [78–80].

The concept of student engagement defies a singular definition, as it encapsulates a complex interplay of factors [81]. Engaged students are characterized by a sense of purpose, persistence, resilience, and an emotional connection to their learning environment. They experience a deep sense of belonging and harbor a high degree of self-efficacy, factors that contribute to their multifaceted engagement [84]. It’s important to recognize that student engagement is not static; rather, it evolves over time within the realm of an individual’s self-determination [85]. Acknowledging the multidimensionality and dynamism of engagement, universities are called upon to take a proactive stance in promoting and fostering student engagement in the context of online learning, ensuring not only academic success but also the holistic well-being of their students.

To effectively meet the challenges posed by the shift to online learning, universities need to provide comprehensive support mechanisms for students [76, 77]. This support takes various forms, ranging from mental health counseling to personalized academic advising and the establishment of online peer support groups. Recognizing the unique stresses and hurdles that come with remote learning, institutions must proactively address the well-being of their students, fostering an environment that not only cultivates engagement but also offers the necessary resources to cope with the challenges that this mode of learning entails. By doing so, universities can proactively contribute to the academic success and overall well-being of their student body in an evolving educational landscape.

## Conclusions

Engagement and several positive psychological traits play a pivotal role in contributing to students’ overall educational attainment. These favorable psychological attributes and experiences demonstrate a substantial positive influence on academic accomplishments, learning experiences, and attrition rates [91–94]. Academic resilience, denoting the capacity to adeptly confront academic challenges, displays a favorable association with academic achievement. Similarly, academic motivation, characterized by the ability to rebound from academic setbacks and sustain academic performance, correlates positively with academic achievement, Grade Point Average (GPA), and reduction of test anxiety. Moreover, academic well-being, encompassing the positive psychological state associated with academic contentment and achievement, exhibits a constructive connection with academic attainment, learning experiences, and inversely with dropout rates. Additionally, the phenomenon of learner enjoyment, encompassing positive emotions and pleasure derived from learning endeavors, manifests a positive correlation with academic achievement and motivation.

A comprehensive comprehension of the role played by these affirmative psychological traits and experiences in students’ educational accomplishment empowers educators to formulate strategies that foster the cultivation of these vital proficiencies [92, 93]. Propagating optimistic academic attitudes and behaviors, such as resilience, self-efficacy, and positive emotions, emerges as a viable strategy for augmenting students’ academic performance and well-being. By establishing a supportive pedagogical milieu and providing pertinent educational resources, educators facilitate the development of the requisite competencies for academic success and the enjoyment of the learning process. In sum, the findings proffer the notion that an all-encompassing approach to education, one that factors in students’ affirmative psychological traits and experiences, holds promise for optimizing students’ educational achievement. By endorsing academic resilience, diverse personality traits, academic well-being, and robust student engagement, educators can effectively propel students toward their utmost potential and academic triumph [94].

The contributions of academic resilience, personality, academic well-being, and educational attainment to online instruction are multifaceted and vital in shaping the landscape of digital education. Academic resilience plays a pivotal role in helping students navigate the challenges of online learning, providing them with the mental fortitude to overcome obstacles and persevere in their academic pursuits. Personality traits, such as extraversion, openness, and conscientiousness, influence how students engage with online content, interact with peers, and manage their time effectively in the virtual learning environment. Academic well-being, encompassing emotional and psychological aspects, greatly impacts students’ overall satisfaction and motivation in online classes, contributing to their engagement and persistence. Educational attainment, a primary goal of online instruction, is influenced by these factors, as they collectively determine students’ ability to succeed academically and meet their learning objectives in the digital realm. Understanding the intricate interplay of these factors is essential for educators, institutions, and policymakers to develop effective online learning strategies, provide tailored support, and enhance the quality of online education for diverse student populations.

### Implications

The study yields significant theoretical and pedagogical implications for the realm of online education. The theoretical insights emphasize the integration of resilience-building strategies into curricula, positioning resilience as a fundamental component of effective education. This suggests that educational institutions should prioritize the development of adaptable coping mechanisms and growth mindsets to empower students in navigating the challenges of online learning. Additionally, the study underscores the importance of a holistic approach to education, emphasizing the pivotal role of academic well-being in shaping student engagement and educational attainment. This theoretical framework highlights the interconnectedness of psychological well-being and academic success, advocating for mental health support, stress reduction strategies, and work-life balance within educational environments.

Furthermore, the theoretical implications stress the significance of student engagement in educational attainment, urging educators to embrace diverse teaching methodologies and interactive online spaces to foster engagement and enhance the overall learning experience. The study’s recognition of the role of personality traits in educational contexts underscores the importance of personality education within higher education. Encouraging the development of positive personality traits, emotional intelligence, and interpersonal skills becomes crucial in empowering students for success in both online and offline learning environments.

The pedagogical implications of the study provide actionable recommendations for educators and institutions. Firstly, educators are encouraged to incorporate resilience-building strategies into their curricula, offering workshops and resources that address stress management and problem-solving skills. Prioritizing student well-being through mental health support, stress reduction strategies, and work-life balance initiatives becomes imperative for institutions. Diverse teaching methodologies that facilitate collaboration, interaction, and practical application should be embraced to enhance student engagement and the overall learning experience. Additionally, personalized feedback aligned with different personality profiles should be considered, optimizing engagement and attainment.

### Limitations and recommendations for further studies

This study possesses several limitations that warrant consideration for future research endeavors. Firstly, the sample specificity poses a limitation, as the study focused exclusively on university students in China using the Tencent Meeting application for online classes. Consequently, the findings may lack generalizability to other online learning platforms or diverse student populations in different cultural or geographical contexts. To address this, future research should adopt a more diverse and inclusive approach to encompass a wider array of online learning settings and student demographics. Secondly, the cross-sectional design employed in this study, which captured data at a single point in time, restricts our understanding of the dynamic nature of academic resilience, well-being, personality traits, and their evolving impact on educational attainment in online classes. To overcome this limitation, longitudinal studies are recommended, offering insights into the trajectories and developmental aspects of these variables over an extended duration.

Another notable limitation pertains to the reliance on self-report measures for data collection. This approach may introduce response bias and social desirability effects. Future research would benefit from incorporating objective measures or multiple data sources to enhance the validity and reliability of the findings. Furthermore, the study primarily focused on specific personality traits, namely extraversion, openness, and conscientiousness. Expanding the scope to encompass a broader spectrum of personality factors could offer a more comprehensive understanding of their influence on online learning.

As for areas for future studies, a comparative analysis of online education outcomes across various digital platforms, beyond Tencent Meeting, could uncover platform-specific effects on academic resilience, well-being, personality, student engagement, and educational attainment. Additionally, exploring how cultural variations impact the relationships among these variables in online education is essential, warranting a comparative cross-cultural investigation.

In addition, longitudinal research tracking students’ academic journeys in online classes over an extended period can provide valuable insights into the development and changes in academic resilience, well-being, personality traits, and their effects on educational attainment. Moreover, examining the effectiveness of interventions designed to enhance academic resilience, promote well-being, or address specific personality traits in online learners could offer practical insights for educators and institutions striving to improve student success. Also, to gain a more comprehensive understanding of the complex interplay among these variables in online education settings, a mixed-methods approach, combining quantitative data with qualitative insights, could prove invaluable. Furthermore, future research should delve into the inclusivity and accessibility of online education platforms, particularly concerning students with diverse needs, disabilities, or those facing digital access challenges. Lastly, investigating the impact of educational policies and institutional practices on the variables studied can help identify systemic factors influencing student engagement and attainment in online classes. Additionally, exploring the role of emerging technologies, such as artificial intelligence or virtual reality, in mitigating challenges and enhancing student engagement and success in online education presents a promising avenue for future investigation.

## Data Availability

The data will be made available upon request from the corresponding author (Corresponding author: e-mail: ll5618@hpu.edu.cn).
